# Synthesis and Excellent Duplex Stability of Oligonucleotides Containing 2′-Amino-LNA Functionalized with Galactose Units

**DOI:** 10.3390/molecules22050852

**Published:** 2017-05-21

**Authors:** Rajesh Kumar, Annika Ries, Jesper Wengel

**Affiliations:** 1 Biomolecular Nanoscale Engineering Center, Department of Physics, Chemistry and Pharmacy, University of Southern Denmark, Campusvej 55, 5230 Odense M, Denmark; rajeshenzyme@gmail.com (R.K.); ries.annika@web.de (A.R.); 2 Department of Chemistry, University of Delhi, Delhi 110007, India

**Keywords:** 2′-Amino-LNA, click chemistry, duplex stability, triantennary galactose ligand, oligonucleotides

## Abstract

A convenient method for the preparation of oligonucleotides containing internally-attached galactose and triantennary galactose units has been developed based on click chemistry between 2′-*N*-alkyne 2′-amino-LNA nucleosides and azido-functionalized galactosyl building blocks. The synthesized oligonucleotides show excellent binding affinity and selectivity towards complementary DNA/RNA strands with an increase in the melting temperature of up to +23.5 °C for triply-modified variants.

## 1. Introduction

**Figure 1:**
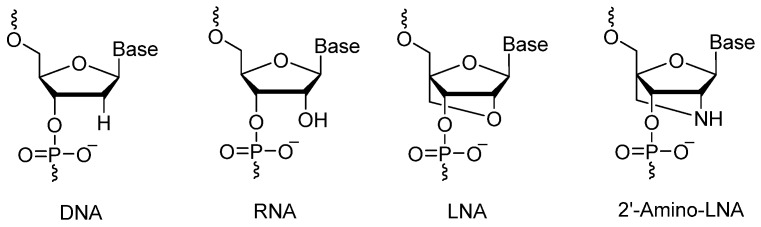
Chemical structure of DNA, RNA, LNA, and 2′-amino-LNA nucleotide monomers.

Nucleic acids have attracted ample interest in the areas of nanotechnology, biology, and biochemistry over the past decade [1,2,3,4]. Oligonucleotide (ON) chemistry has been developed with the aim of modifying the sugar ring [5,6], the nucleobases [7,8,9], or the backbone [10,11], and numerous studies have highlighted the importance of the furanose ring conformation on the biophysical properties of ONs [12,13,14,15,16,17]. Locked nucleic acid (LNA, Figure 1) [18,19] is a very promising candidate of this class of compounds as the incorporation of a LNA monomer into ONs has been reported to induce excellent duplex stability, favorable mismatch discrimination, and improved resistance to nucleolytic digestion [20,21,22,23,24]. The bicyclic skeleton which “locks” the furanose ring of LNA nucleotides into an RNA mimicking C3′-*endo* (*N*-type) conformation is key to these specific properties making LNA-containing oligonucleotides an attractive tool for diagnostic and therapeutic applications [25,26,27,28,29]. As a close analog to LNA, 2′-amino-LNA (Figure 1) [30,31] has been developed to enable incorporation of additional functional groups along the minor groove of high-affinity ONs [32], and recently it was shown that incorporation of 2′-amino-LNA monomers can improve the therapeutic potential of siRNA and aptamer constructs [33,34,35]. 

Several research groups have during the last years been involved in the synthesis of ONs conjugated with galactose (Gal) and *N*-acetylgalactosamine (GalNAc) units as asialoglycoprotein receptor (ASGPR) ligands [36,37,38,39,40,41,42,43]. As one example, GalNAc-conjugated siRNAs targeting TTR (transthyretin) mRNA has been reported to show potent activity in human liver and to reduce circulating TTR protein levels in the blood [44]. Likewise, Østergaard et al. [45,46] demonstrated that 5′-conjugated triantennary GalNAc antisense oligonucleotides (ASOs) act as a hepatocyte targeting pro-drug which can be metabolized to liberate the parent ASO inside liver cells, and similar targeting has been reported using conjugated siRNAs [47,48,49].

Among multiple DNA linking strategies developed to date, the copper(I)-catalyzed azide alkyne cycloaddition (CuAAC) reaction is a robust and highly adaptable bioconjucation process (“click reactions”) for ONs allowing preparation of various conjugates in high yields [50,51,52,53], and single to triple modification of oligonucleotides by this reaction has been reported [54,55,56,57,58,59]. 

Herein, we report synthesis and characterization of ONs modified via copper(I)-catalyzed click chemistry with galactose derivatives **M^2^** and **M^3^**. 

## 2. Results and Discussion

### 2.1. Synthesis of Azido-Functionalized Galactose Derivatives

**Scheme 1:**
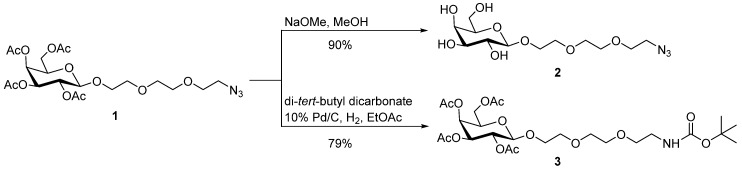
Synthesis of galactopyranoside intermediates.

2-[2-(2-Azidoethoxy)ethoxy]ethoxy-2,3,4,6-tetra-*O*-acetyl-β-d-galactopyranoside (**1**) and 2-[2-(2-azidoethoxy)ethoxy]ethoxy-β-d-galactopyranoside (**2**) were synthesized according to procedures in the literature [60,61]. For synthesis of compound **3** we first tried the reduction of azido group **1** into an amino group with 10% Pd/C in different organic solvents (MeOH/EtOH/EtOAc) in an H_2_ atmosphere. We observed in every case intramolecular acetyl migration from the sugar to the amine. To avoid this acetyl migration the amine was directly equipped with a Boc-protecting group after azide reduction with Pd/C in EtOAc furnishing galactopyranoside **3** in 79% yield (Scheme 1). 

**Scheme 2:**
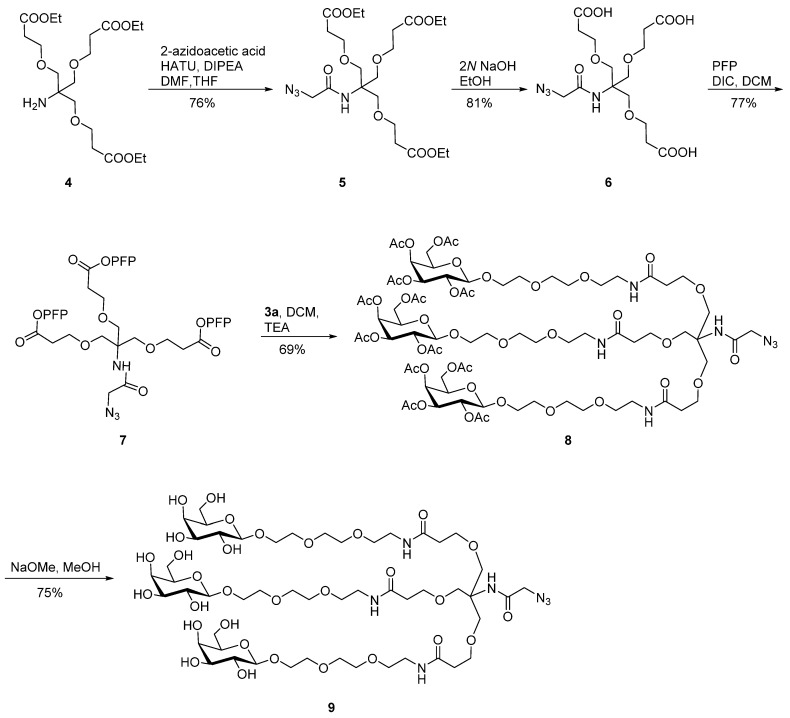
Synthesis of triantennary azido galactopyranoside.

The amino group of compound **4** [62] was subsequently reacted with 2-azidoacetic acid in *N*,*N*-dimethylformamide/tetrahydrofuran (DMF/THF) in the presence of 1-[bis(dimethylamino)methylene]-1*H*-1,2,3-triazolo-[4,5-*b*]pyridinium 3-oxide hexafluorophosphate (HATU) and *N*,*N*-diisopropylethylamine (DIPEA) to afford azido derivative **5** in 76% yield. The ester groups were next converted into the corresponding acids to give **6**, followed by reaction with pentafluorophenol (PFP) in dichloromethane (DCM) in the presence of *N*,*N*-diisopropylcarbodiimide (DIC) to afford tris-pentafluorophenol ester **7** in 77% yield. The Boc-protecting group of galactopyranoside **3** was removed by standard conditions (1:3 trifluoroacetic acid (TFA)/DCM, 1 h) yielding the corresponding amino galactopyranoside intermediate **3a**. Further reaction with tris-pentafluorophenol ester **7** furnished the triantennary compound **8** in 69% yield, which upon ester cleavage was converted into the desired triantennary azido galactopyranoside **9** in 75% yield (Scheme 2).

### 2.2. ON Synthesis

**Scheme 3:**
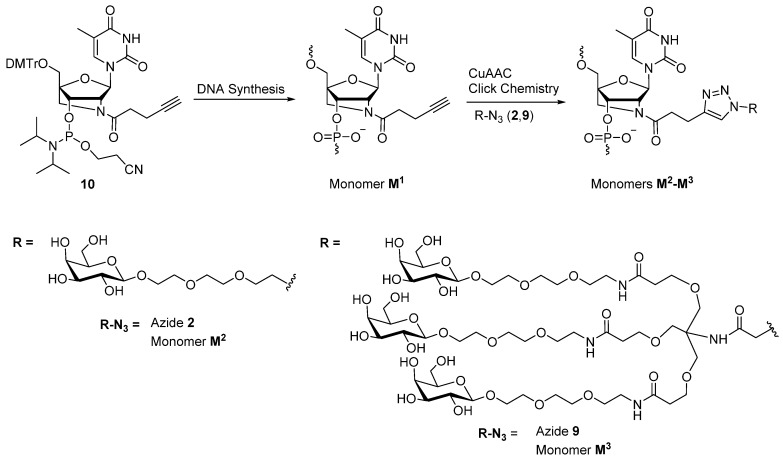
Synthesis of alkynyl 2′-amino-LNA monomer **M^1^** and incorporation of monomers **M^2^** and **M^3^** into ONs.

Synthesis of 9-mer ONs was performed at a 1.0 μmol scale using standard solid-phase phosphoramidite chemistry on an automated DNA synthesizer. Phosphoramidite building block **10** [54] showed a satisfactory step-wise coupling yield of >94% using 1*H*-tetrazole as the activator and a coupling time of 20 min used for incorporation of monomer **M^1^** into ONs, one or three times according to Lou et al. [63] to afford **ON1** (5′-GTGA**M^1^**ATGC) and **ON2** (5′-G**M^1^**GA**M^1^**A**M^1^**GC) (Scheme 3). The synthesized ONs were passed through NAP-10 column, and their purity (>90%) and composition was confirmed by ion-exchange (IE) HPLC and MALDI-MS (see Section 3.8), respectively.

### 2.3. Post-Oligomerization CuAAC Click Chemistry

Post-oligomerization (i.e., after completion of ON synthesis and purification) azide-alkyne click chemistry on **ON1** and **ON2** was accomplished with mono and triantennary azido galactopyranosyl derivatives **2** and **9** under microwave conditions. Dimethyl sulfoxide (DMSO), 2 Mtriethylammonium acetate buffer (pH 7.4), azides **2** or **9**, copper(II)sulfate-tri(benzyltriazolylmethyl)amine (TBTA) (1:1) and ascorbic acid were added to a deaerated aqueous solution of the starting ON. The purity (>90%) and composition of the products **ON3**–**ON6** was confirmed by IE HPLC and MALDI-MS (see Section 3.10), respectively. 

### 2.4. Thermal Denaturation Studies

#### 2.4.1. Binding Affinity

The effect upon incorporation of one (**ON3** and **ON5**) or three (**ON4** and **ON6**) galactopyranosyl modifications into mixed sequence 9-mer ONs on the thermal stability and base-pairing specificity of duplexes with DNA and RNA complements was evaluated by UV-VIS thermal denaturation experiments (Table 1 and Table 2). All changes in thermal denaturation temperatures (*T*_m_) of modified nucleic acid duplexes are discussed relative to the *T*_m_ values measured for the unmodified reference duplexes.

**ON7** and **ON8** containing one or three non-conjugated 2′-amino-LNA-T monomers showed a substantially increased thermal affinity towards DNA (Δ*T*_m_ value +9.5 °C with three monomers **M^4^**) and RNA complementary strands (Δ*T*_m_ value +20.0 °C with three monomers **M^4^**) (Table 1). Remarkably, the corresponding ONs containing one or three monomer(s) **M^2^** or **M^3^** (**ON3**–**ON6**) display similar increases in thermal affinity, with **ON5**:DNA (single incorporation of **M^3^**) as the only exception (Δ*T*_m_ value of only +2.0 °C (Table 1)). These results demonstrate that both the mono- and triantennary galactopyranosyl unit are well tolerated in a short ON aimed at DNA or RNA targeting. 

#### 2.4.2. Binding Specificity

Next, the binding specificities of single- and triple-modified oligonucleotides were evaluated against centrally-positioned mismatched bases (Table 2). We found, in general, very similar pairing selectivity for all sequences (**ON^ref^**, **ON3**–**ON8**) with the notable exceptions that the modified strands all showed better discrimination against a DNA or RNA G mismatch than the control, and that the control showed better discrimination against an RNA C mismatch than the modified sequences. 

All DNA **ON^ref^** displayed a thermal denaturation temperature of 29.0 °C with a DNA complementary strand, and an RNA counterpart at 27.0 °C. The results of **ON3** showed that the monoantennary galactopyranosyl functionalized triazole-linked 2′-amino-LNA was well tolerated in a DNA/DNA duplex as the thermal denaturation temperature was comparable with that of **ON^ref^** (Table 1). A remarkable stabilization at 8.5 °C was observed with the RNA complement. The corresponding three monoantennary galactopyranosyl units in **ON4** was better tolerated as the thermal denaturation temperature was induced by 11.0 °C against a DNA complement and 23.5 °C against an RNA complement. In general, similar trends were observed in **ON5**–**ON8** showed increased thermal duplex stability against both DNA and RNA relative to **ON^ref^**, but the sequence containing a singly-incorporated triantennary galactopyranosyl unit in **ON5** stabilized less than the non-conjugated 2′-amino-LNA **ON7**. Additionally, the corresponding non-conjugated 2′-amino-LNA (**ON8**) was better tolerated than the triply-incorporated triantennary galactopyranosyl unit in **ON6** against a DNA complement, and a reverse trend was observed in an RNA complement, respectively (Table 1). For evaluation of the mismatch discriminative properties of these mono-/triantennary galactopyranosyl-conjugated and non-conjugated 2′-amino-LNA ONs were chosen with one mismatched nucleotide opposite the sugar modification. All modifications showed similar, or even enhanced, mismatch discrimination relative to the unmodified strand **ON^ref^**, with the discriminative power generally superior against DNA strands (Table 2).

In summary, singly- or triply-incorporated galactopyranose moieties induced significantly increased duplex stability relative to the reference duplexes, thus expanding the results reported for galactopyranose-modified carbohydrate moieties attached to the 2′-position of nucleotides via triazole [64] and amide linkages [65,66]. The data reported herein, in combination with the remarkable exonuclease resistance induced by other *N*-functionalized 2′-amino-LNA nucleotides [63,65], establish monomers **M^2^** and **M^3^** as promising constituents of future RNA-targeting oligonucleotide drugs.

## 3. Materials and Methods

### 3.1. General Information

All reagents were purchased from Sigma-Aldrich (St. Louis, MO, USA) and used without further purification. Dichloromethane, *N*,*N*-diisopropylethylamine (DIPEA), THF, methanol, *N*,*N*-dimethylformamide (DMF) and pyridine were dried over activated 4 Å molecular sieves, and petroleum ether (PE) and ethylacetate (EtOAc) were used as received. All reactions conducted in anhydrous solvents were carried out under an argon atmosphere. Silica gel column chromatography was performed using Merck Millipore silica gel 60 (0.040–0.063 mm) (Darmstadt, Germany). Thin layer chromatography (TLC) was performed using Merck silica gel 60 F254 (0.22 mm thickness, aluminum-backed) (Darmstadt, Germany). Compounds were visualized at 254 nm or stained with 5% sulfuric acid in EtOH. ^1^H-NMR spectra were measured at 400 MHz, ^13^C-NMR spectra were measured at 101 MHz, and ^19^F-NMR spectra were measured at 376 MHz, all on a Bruker AVANCE III 400 spectrometer (Billerica, MA, USA). Chemical shifts are given in ppm and *J* values are given in Hz. All assignments for ^1^H-NMR and ^13^C-NMR have been confirmed by COSY, HSQC, and HMBC. HRMS-ESI spectra were recorded on a Bruker APEX III FT-ICR mass spectrometer (Billerica, MA, USA).

### 3.2. Synthesis of 2-[2-(2-Tert-butyloxycarbonylamidoethoxy)ethoxy]ethoxy 2,3,4,6-tetra-O-acetyl-*β*-d-galactopyranoside *(**3**)*

2-[2-(2-Azidoethoxy)ethoxy]ethoxy-2,3,4,6-tetra-*O*-acetyl-β*-*d-galactopyranoside (**1**, 500 mg, 0.99 mmol) was dissolved in EtOAc (5.0 mL), and 10% Pd/C (6.0 mg) and di-*tert*-butyl dicarbonate (324 mg, 1.48 mmol) were added. The reaction mixture was stirred at r.t. in an H_2_ atmosphere for 6 h according to a literature procedure [67]. The reaction mixture was passed through a celite pad and concentrated to dryness under reduced pressure. The residue was purified by silica gel column chromatography using 20–40% EtOAc in petroleum ether (*v*/*v*) as eluent to afford compound **3** as a viscous oil (451 mg) in 79% yield. *R*_f_ = 0.3 (EtOAc/PE, 50:50). ^1^H-NMR (400 MHz, CDCl_3_): *δ* 5.39 (d, *J* = 2.6 Hz, 1H, H-4), 5.21 (dd, *J* = 10.5, 8.0 Hz, 1H, H-2), 5.02 (dd, *J* = 10.4, 3.4 Hz, 1H, H-3), 4.56 (d, *J* = 8.0 Hz, 1H, H-1), 4.22–4.07 (m, 2H, H-6a, H-6b), 4.01–3.88 (m, 2H, H-5, CH_2_), 3.78–3.72 (m, 1H, CH_2_), 3.71–3.58 (m, 7H, CH_2_, NH), 3.54 (t, *J* = 5.2 Hz, 2H, CH_2_), 3.33–3.30 (m, 2H, CH_2_), 2.15 (s, 3H, OAc), 2.06 (s, 3H, OAc), 2.05 (s, 3H, OAc), 1.99 (s, 3H, OAc), 1.45 (s, 9H, t-Bu). ^13^C-NMR (101 MHz, CDCl_3_): *δ* 170.4, 170.2, 170.1, 169.5 (4 × CH_3_COO), 156.0 (NHCO), 101.4 (C-1), 79.2 (C(CH_3_)_3_), 70.9 (C-2), 70.7 (C-3), 70.3, 69.1 ((OCH_2_CH_2_)_2_OCH_2_), 68.8 (C-5), 67.1 (C-4), 61.3 (C-6), 40.4 (CH_2_NH), 28.4 (C(CH_3_)_3_), 20.7 (CH_3_COO), 20.7 (2 × CH_3_COO), 20.6 (CH_3_COO). HRMS (ESI) calcd. for [C_25_H_41_NO_14_Na]^+^: 602.2419; found: 602.2416.

### 3.3. Synthesis of 2-Azidoacetyl-N-{tris[3-(ethylcarboxylethoxy)methyl]methyl}-amine *(**5**)*

To a solution of amine **4** [62] (1.0 g, 2.37 mmol) and 2-azidoacetic acid (310 mg, 3.06 mmol) in THF (30 mL) at r.t. were added DIPEA (0.54 mL, 3.08 mmol) and HATU (1.17 g, 3.07 mmol in 10 mL DMF). The reaction mixture was stirred for 6 h whereupon EtOAc (50 mL) was added. Washings were performed using saturated aq. NaHCO_3_ (3 × 50 mL) and brine (3 × 50 mL), and the organic phases were dried (Na_2_SO_4_), filtered, and evaporated to dryness under reduced pressure. The residue was purified by silica gel column chromatography 0–2% MeOH in CH_2_Cl_2_ (*v*/*v*) as eluent to afford compound **5** as a viscous oil (0.91 g) in 76% yield. *R*_f_ = 0.6 (MeOH/CH_2_Cl_2_, 5:95). ^1^H-NMR (400 MHz, CDCl_3_): *δ* 6.56 (brs, 1H, NH), 4.15 (q, *J* = 7.2 Hz, 6H, CH_2_), 3.85 (s, 2H, CH_2_), 3.78–3.62 (m, 12H, CH_2_), 2.54 (t, *J* = 6.2 Hz, 6H, CH_2_), 1.27 (t, *J* = 7.1 Hz, 9H, CH_3_). ^13^C-NMR (101 MHz, CDCl_3_): *δ* 171.6 (CO_2_Et), 166.7 (CONH), 69.0 (CH_2_), 66.8 (CH_2_), 60.5 (CH_2_), 59.9 (CH_2_), 52.8 (C(CH_2_)_3_), 35.0 (CH_2_), 14.2 (CH_3_). HRMS (ESI) calcd. for [C_21_H_36_N_4_O_10_Na]^+^: 527.2324; found: 527.2320. 

### 3.4. Synthesis of 2-Azidoacetyl-N-{tris[(2-carboxyethoxy)methyl]methyl}amine *(**6**)*

Compound **5** (500 mg, 1.19 mmol) was dissolved in ethanol (5.0 mL) and aq. NaOH (2 N, 1 mL) was added. The reaction mixture was stirred at r.t. for 6 h, concentrated to half value by evaporation under reduced pressure, adjusted to pH 3 with aq. HCl (1 N, 2 mL), and extracted with EtOAc (100 mL). The organic phase was dried (Na_2_SO_4_), filtered and evaporated to dryness under reduced pressure to afford compound **6** as an off-white solid material (340 mg) in 81% yield. *R*_f_ = 0.5 (MeOH/CH_2_Cl_2_/CH_3_COOH, 2:18:1). ^1^H-NMR (400 MHz, DMSO-*d*_6_): *δ* 12.17 (brs, 3H, COOH), 7.35 (brs, 1H, NH), 3.76 (s, 2H, CH_2_), 3.67–3.48 (m, 12H, CH_2_), 2.43 (t, *J* = 6.3 Hz, 6H, CH_2_). ^13^C-NMR (101 MHz, DMSO-*d*_6_): δ 172.5 (3 × COOH), 167.2 (CONH), 67.9 (CH_2_O), 66.6 (CH_2_O), 59.9 (C(CH_2_)_3_), 50.7 (CH_2_N_3_), 34.5 (CH_2_COOH). HRMS (ESI) calcd. for [C_15_H_24_N_4_O_10_Na]^+^: 443.1385; found: 443.1400.

### 3.5. Synthesis of 2-Azidoacetyl-N-{tris[3-(pentafluorophenylcarboxylethoxy)methyl]methyl}amine *(**7**)*

Compound **6** (470 mg, 1.12 mmol) was dissolved in CH_2_Cl_2_ (7.0 mL) and pentafluorophenol (1.02 g, 5.59 mmol) was added. The reaction mixture was cooled to 0 °C and *N*,*N′*-diisopropylcarbodimide (0.98 mL, 6.26 mmol) was added, and the reaction mixture was stirred for 6 h at r.t. The solvent was removed under reduced pressure and the residue was purified by silica gel column chromatography using EtOAc/PE (5–20%, *v*/*v*) as eluent to afford compound **7** as a viscous oil (789 mg) in 77% yield. *R*_f_ = 0.5 (EtOAc/PE, 2:3). ^1^H-NMR (400 MHz, CDCl_3_): *δ* 6.47 (brs, 1H, NH), 4.10–3.49 (m, 14H, CH_2_O, CH_2_N_3_), 2.91 (t, *J* = 5.9 Hz, 6H, CH_2_COO). ^13^C-NMR (101 MHz, CDCl_3_): *δ* 167.5 (COOPFP), 166.9 (COCH_2_N_3_), 142.3 (Ar-C), 139.8 (Ar-C), 139.8–138.7 (Ar-C), 136.6 (Ar-C), 68.9 (CH_2_O), 66.1 (CH_2_O), 59.8 (C(CH_2_)_3_), 52.8 (CH_2_N_3_), 34.2 (CH_2_COOPFP). ^19^F-NMR (376 MHz, CDCl_3_): *δ* −152.9 (dd, *J* = 21.7, 4.2 Hz, 6F), −157.8 (t, *J* = 21.7 Hz, 3F), −162.09–−162.64 (m, 6F). HRMS (ESI) calcd. for [C_33_H_21_F_15_N_4_O_10_Na]^+^: 941.0910; found: 941.0910.

### 3.6. Synthesis of Triantennary Azido 2,3,4,6-Tetra-O-Acetyl-*β*-d-Galactopyranoside *(**8**)*

Compound **3** (530 mg, 0.91 mmol) was dissolved in CH_2_Cl_2_/trifluoroacetic acid (3:1, 12 mL) and stirred at r.t. for 1 h. The solvents were evaporated under reduced pressure and the resulting oil was dissolved in anhydrous CH_2_Cl_2_ (24 mL). To this mixture was added *N*-{tris[3-(pentafluorophenylcarboxylethoxy)methyl]methylamide}-2-azidoacetamide (**7**, 210 mg, 0.23 mmol) and the resulting mixture was adjusted to pH 8 with triethylamine (TEA) and stirred at r.t. for 10 h. The solvents were removed under reduced pressure and the residue was redissolved in CH_2_Cl_2_ (50 mL) and washed with H_2_O (3 × 50 mL). The separated organic phase was dried (Na_2_SO_4_), filtered and evaporated to dryness under reduced pressure. The residue was purified by silica gel chromatography using (MeOH/CH_2_Cl_2_, 5:95 *v*/*v*) as eluent to afford the acetyl protected triantennary galactose azide **8** as a white solid material (285 mg) in 69% yield. *R*_f_ = 0.4 (MeOH/CH_2_Cl_2_, 5:95). ^1^H-NMR (400 MHz, CDCl_3_): *δ* 6.90 (brs, 1H, NH), 6.59 (t, *J* = 5.5 Hz, 3H, NH), 5.39 (d, *J* = 3.1 Hz, 3H, Gal-H-4), 5.20 (dd, *J* = 10.4, 8.0 Hz, 3H, Gal-H-2), 5.03 (dd, *J* = 10.5, 3.4 Hz, 3H, Gal-H-3), 4.56 (d, *J* = 7.9 Hz, 3H, Gal-H-1), 4.20–4.10 (m, 6H, H-6), 4.01–3.90 (m, 6H, Gal-H-5, CH_2_), 3.88 (s, 2H, CH_2_N_3_), 3.78–3.68 (m, 15H, CH_2_O), 3.68–3.58 (m, 18H, CH_2_O), 3.56 (t, *J* = 5.3 Hz, 6H, CH_2_O), 3.48–3.42 (m, 6H, CH_2_NH), 2.44 (t, *J* = 5.8 Hz, 6H, CH_2_CO), 2.15 (s, 9H, OAc), 2.06 (s, 9H, OAc), 2.05 (s, 9H, OAc), 1.99 (s, 9H, OAc). ^13^C-NMR (101 MHz, CDCl_3_): *δ* 171.2, 170.4, 170.2, 170.1 (4 × CH_3_COO), 169.5 (CH_2_CONH), 167.2 (COCH_2_N_3_), 101.4 (Gal-C-1), 70.9 (Gal-C-2), 70.7 (Gal-C-3), 70.6, 70.3, 70.1, 69.9, 69.3, 69.1, 67.1 (7 × CH_2_O), 68.9 (Gal-C-5), 67.5 (Gal-C-4), 61.2 (Gal-C-6), 60.1 (C(CH_2_)_3_), 52.5 (CH_2_N_3_), 39.3 (CH_2_NH), 36.6 (CH_2_CO), 20.8, 20.64, 20.62, 20.55 (4 × CH_3_COO). HRMS (ESI) calcd. for [C_75_H_117_N_7_O_43_Na]^+^: 1826.7076; found: 1826.7052.

### 3.7. Synthesis of Triantennary Azido 2-*β*-d-Galactopyranoside *(**9**)*

Acetyl protected triantennary galactose azide **8** (140 mg, 0.08 mmol) was dissolved in anhydrous MeOH (20 mL) and NaOMe (25 wt. % in methanol, 4 μL, 8.0 μmol) was added. The reaction mixture was stirred at r.t. for 1 h, neutralized with DOWEX-50WX2 (H^+^ resin), filtered, and evaporated to dryness under reduced pressure. The residue was redissolved in H_2_O, dialyzed for two days using Spectra/Por Float-A-Lyzer G2 (MWCO: 100–500 D molecular weight cut-off) and lyophilized to furnish the desired product **9** as a white solid material (76 mg) in 75% yield; *R*_f_ = 0.4 (CH_2_Cl_2_/MeOH/H_2_O, 1.6:1.0:0.2). ^1^H-NMR (400 MHz, MeOD-*d*_4_): *δ* 4.30 (d, *J* = 7.3 Hz, 3H, Gal-H-1), 4.12–3.99 (m, 3H), 3.9–3.87 (m, 6H), 3.78–3.67 (m, 40H), 3.60–3.50 (m, 17H), 3.42 (t, *J* = 5.1 Hz, 6H, CH_2_NH), 2.49 (t, *J* = 5.7 Hz, 6H, CH_2_CO). ^13^C-NMR (101 MHz, MeOD-*d*_4_): *δ* 174.2 (CH_2_CONH), 170.0 (COCH_2_N_3_), 105.0 (Gal-C-1), 76.7, 74.9, 72.5 (Gal-C-2, Gal-C-3, Gal-C-5), 71.5, 71.2, 70.7, 70.4, 70.1, 69.6 (6 × CH_2_O), 68.8 (Gal-C-4), 62.6 (C(CH_2_)_3_), 61.8 (Gal-C-6), 53.2 (CH_2_N_3_), 40.5 (CH_2_NH), 37.5 (CH_2_CO); HRMS (ESI) calcd. for [C_51_H_93_N_7_O_31_Na]^+^: 1322.5808; found: 1322.5846.

### 3.8. Synthesis and Purification of ON1–ON2

ONs were synthesized on a DNA synthesizer (PerSpective Biosystems Expedite 8909, (Framingham, MA, USA)) in 1.0 μmol scale using manufacturer’s standard protocols. For incorporation of monomer **M^1^** [56] a hand-coupling procedure [68] was used (20 min coupling time and 5-[3,5-bis(trifluoromethyl)phenyl]-1*H*-tetrazole (0.25 M, in anhydrous CH_3_CN) as activator). The coupling efficiencies of standard DNA phosphoramidites and phosphoramidite **10** based on the absorbance of the dimethoxytrityl cation released after each coupling varied between 95% and 98%. Cleavage from solid support and removal of nucleobase protecting groups was performed using 32% aq. ammonia for 16 h at 55 °C. The resulting oligonucleotides were purified by DMT-ON RP-HPLC using Waters System 600 (Milford, MA, USA) equipped with Xterra MS C18-column (5 μm, 150 mm × 7.8 mm, (Milford, MA, USA)). Elution was performed starting with an isocratic hold of A-buffer for 5 min followed by a linear gradient to 70% B-buffer over 40 min at a flow rate of 1.0 mL/min (A-buffer: 0.05 M triethyl ammonium acetate, pH 7.4; B-buffer: 25% buffer A, 75% CH_3_CN). RP-purification was followed by detritylation (80% aq. AcOH, 30 min) and precipitation (acetone, −18 °C, 12 h). The identity and purity of **ON1** and **ON2** were verified by MALDI-TOF mass spectrometry (Billerica, MA, USA) (Table 3) and IE HPLC, respectively. IE HPLC was performed using a Merck Hitachi LaChrom instrument (Tokyo, Japan) equipped with a Dionex DNAPac Pa-100 column (250 mm × 4 mm, Sunnyvale, CA, USA). Elution was performed starting with an isocratic hold of A- and C-buffers for 2 min followed by a linear gradient to 60% B-buffer over 28 min at a flow rate of 1.0 mL/min (A-buffer: Milli-Q water; B-buffer: 1 M NaClO_4_, C-buffer: 25 mM Tris-Cl, pH 8.0). MALDI-TOF mass-spectrometry was performed using a MALDI-LIFT system on the Ultraflex II TOF/TOF instrument from Bruker (Billerica, MA, USA) using an HPA-matrix (10 mg 3-hydroxypicolinic acid, 50 mM ammonium citrate in 70% aq. CH_3_CN).

### 3.9. Thermal Denaturation Studies

Thermal denaturation studies were carried out on a Perkin Elmer Lambda 35 UV-VIS spectrometer (Shelton, CT, USA) using a Hellma SUPRASIL synthetic quartz 10 mm path length cuvettes. The oligonucleotides (1.0 μM per strand) were dissolved in medium salt buffer [NaCl (100 mM), EDTA (0.1 mM), NaH_2_PO_4_ (10 mM), Na_2_HPO_4_ (5 mM), pH 7.0] and the resulting solution heated to 90 °C for 10 min and then slowly cooled down to 5 °C. Concentrations of oligonucleotides were calculated using the extinction coefficients. UV absorbance at 260 nm as a function of time was recorded and the thermal denaturation temperatures (*T*_m_) were determined as the maxima of the first derivative of the UV_260_ vs. temperature curves. The *T*_m_ values are given as averages of two measurements within ±0.5 °C.

### 3.10. Postsynthetic Click Procedure in Solution

ON **ON1** or **ON2** (40 nmol) was dissolved in fresh MQ water (190 μL for one incorporation and 151 μL for three incorporations) in a 1.0 mL microwave vial, and 2 M tetra triethylammonium acetate buffer (pH 7.4; 20 μL), DMSO (84 μL for one incorporation and 52 μL for three incorporations), the corresponding azide **2** or **9** (16 μL (**ON1**) for one incorporation and 48 μL (**ON2**) for three incorporations of 10 mM solution in DMSO), CuSO_4_-TBTA equimolar complex (10 μL of 10 mM stock solution) and ascorbic acid (10 μL of 25 mM freshly prepared stock solution) were subsequently added under an argon atmosphere. The resulting mixture was deaerated, tightly closed, and subjected to microwave conditions (microwave reactor, 60 °C, 2 h, Biotage initiator microwave synthesizer). Thereafter, the reaction was cooled to r.t. and then filtrated through an IIIustra NAP-10 column following manufacture’s protocol. The resulting solution was evaporated to dryness at r.t. under a nitrogen atmosphere. The resulting conjugates **ON3**–**ON6** were analyzed by MALDI TOF mass spectrometry (Table 4) and IE HPLC. The ONs (**ON4** and **ON6**) were further purified by RP HPLC. The yields of the click reactions were determined based on the absorbance of the ONs at 260 nm: 92% (**ON3**), 20% (**ON4**), 77% (**ON5**), and 17% (**ON6**).

## 4. Conclusions

In summary, we have successfully developed the copper(I)-catalyzed azide alkyne cycloaddition click chemistry conjugation of mono- and triantennary galactospyranosyl units to internally-positioned amino-LNA nucleotides. This method is efficient and provides the desired products in a remarkable purity. High-affinity recognition of complementary DNA and RNA strands has been demonstrated, thus confirming the suitability of the N2′-position 2′-amino-LNA nucleotides for attachment of targeting ligands for therapeutic oligonucleotide constructs.

## Figures and Tables

**Table 1:** Thermal denaturation temperatures of matched duplexes (DNA/DNA and DNA /RNA) *^a^.*

ON	Sequence	DNA: 3′-CACTATACG *T*_m_/Δ*T*_m_ (°C)	RNA: 3′-CACUAUACG *T*_m_/Δ*T*_m_ (°C)
ON^ref^	5′-GTGATATGC	29.0	27.0
ON3	5′-GTGA**M^2^**ATGC	33.0/+4.0	35.5/+8.5
ON4	5′-G**M^2^**GA**M^2^**A**M^2^**GC	40.0/+11.0	50.5/+23.5
ON5	5′-GTGA**M^3^**ATGC	31.0/+2.0	34.5/+7.5
ON6	5′-G**M^3^**GA**M^3^**A**M^3^**GC	37.5/+8.5	48.5/+21.5
ON7	5′-GTGA**M^4^**ATGC	33.0/+4.0	35.0/+8.0
ON8	5′-G**M^4^**GA**M^4^**A**M^4^**GC	38.5/+9.5	47.0/+20.0

*^a^* Thermal denaturation temperature *T*_m_ (°C) (change in *T*_m_ relative to corresponding reference duplex ON^ref^:DNA/RNA, Δ*T*_m_ (°C)). *T*_m_ values measured as the maximum of the first derivatives of the melting curves (A_260_ vs. temperature) using 1.0 μM concentration of complementary strand. UV-VIS thermal denaturation experiments performed in a medium salt buffer [NaCl (100 mM), EDTA (0.1 mM), NaH_2_PO_4_ (10 mM), Na_2_HPO_4_ (5 mM), pH 7.0] with reported *T*_m_ values being averages of at least two measurements within ±0.5 °C. **M^4^** is non-conjugated 2′-amino-LNA-T, **M^3^** is triantennary galactopyranosyl-conjugated triazole-linked 2′-amino-LNA, and **M^2^** is monoantennary galactopyranosyl-conjugated triazole-linked 2′-amino-LNA.

**Table 2:** Thermal denaturation temperatures of mismatched duplexes *^a^*.

ON	Sequence X =	DNA: 3′-CACTXTACG				RNA: 3′-CACUXUACG			
		*T*_m_/°C		Δ*T*_m_/°C		*T*_m_/°C		Δ*T*_m_/°C	
		A	T	G	C	A	U	G	C
ON^ref^	5′-GTGATATGC	29.0	−15.0	−9.5	−16.0	27.0	−16.0	−5.0	−21.0
ON3	5′-GTGA**M^2^**ATGC	33.0	−16.0	−15.0	−18.0	35.5	−17.5	−10.5	−17.5
ON4	5′-G**M^2^**GA**M^2^**A**M^2^**GC	40.0	−15.5	−17.5	−18.0	50.5	−16.5	−11.0	−15.5
ON5	5′-GTGA**M^3^**ATGC	31.0	−16.5	−16.0	−18.0	34.5	−17.5	−10.0	−18.0
ON6	5′-G**M^3^**GA**M^3^**A**M^3^**GC	37.5	−15.5	−15.0	−18.0	48.5	−15.5	−10.0	−15.0
ON7	5′-GTGA**M^4^**ATGC	33.0	−16.0	−14.5	−18.5	35.0	−17.0	−9.0	−17.0
ON8	5′-G**M^4^**GA**M^4^**A**M^4^**GC	38.5	−16.0	−15.0	−18.5	47.0	−15.5	−9.0	−15.5

*^a^* For experimental conditions see Table 1. Δ*T*_m_ is the change in *T*_m_ relative to the fully matched ON^ref^:DNA or ON^ref^:RNA duplex (X = A).

**Table 3:** Sequence and MALDI-MS of modified oligonucleotides **ON1** and **ON2**.

ON	Sequence	Calculated	Found
ON1	5′-GTGA**M^1^**ATGC	2861.18	2858.69
ON2	5′G**M^1^**GA**M^1^**A**M^1^**GC	3075.74	3075.78

**M^1^** = 2′-alkyne-2′-amino-LNA.

**Table 4:** Sequence and MALDI-MS of conjugated **ON3**–**ON6**.

ON	Sequence	Calculated	Found
ON3	5′-GTGA**M^2^**ATGC	3198.33	3198.25
ON4	5′-G**M^2^**GA**M^2^**A**M^2^**GC	4087.19	4087.93
ON5	5′-GTGA**M^3^**ATGC	4160.77	4160.66
ON6	5′-G**M^3^**GA**M^3^**A**M^3^**GC	6974.51	6974.76

**M^3^** is triantennary galactopyranosyl-conjugated triazole-linked 2′-amino-LNA, and **M^2^** is monoantennary galactopyranosyl-conjugated triazole-linked 2′-amino-LNA.
